# MF6‐ADJ: A Non‐Intrusive Adjoint Sensitivity Capability for MODFLOW 6

**DOI:** 10.1111/gwat.70025

**Published:** 2025-09-25

**Authors:** Mohamed Hayek, Jeremy T. White, Katherine H. Markovich, Joseph D. Hughes, Marsh Lavenue

**Affiliations:** ^1^ INTERA Incorporated Fort Collins CO; ^2^ INTERA Incorporated Albuquerque NM; ^3^ INTERA Incorporated Chicago IL; ^4^ INTERA Incorporated Austin TX

## Abstract

Adjoint sensitivity analysis provides an efficient alternative to direct methods when evaluating the influence of many uncertain parameters on a limited number of performance measures in hydrologic and hydrogeologic models. However, most adjoint implementations are “intrusive”, requiring extensive modifications of the forward simulation code. This creates significant development and maintenance burdens that limit broad adoption. To address these needs, we present MF6‐ADJ, a “non‐intrusive” adjoint sensitivity capability for the MODFLOW 6 groundwater flow process that leverages the MODFLOW Application Programming Interface (API) to interact with the forward groundwater flow solution without altering its core code. MF6‐ADJ supports both confined and unconfined flow conditions, structured and unstructured grids, and is compatible with both the Standard and Newton–Raphson solution schemes. It computes sensitivities of a wide range of general performance measures, including hydraulic heads, boundary fluxes, and weighted residuals, with respect to key model parameters such as hydraulic conductivity, storage coefficient, injection/extraction rate, recharge rate, boundary head, and boundary conductance. Sensitivities are computed at each node, enabling fine‐grained diagnostic and calibration analysis. Validation against analytical solutions and the finite‐difference perturbation method confirms excellent agreement, while demonstrating speedups ranging from hundreds to tens of thousands of times depending on grid discretization, since the adjoint state method computes sensitivities efficiently at the grid‐block level. This non‐intrusive design makes MF6‐ADJ highly accessible and maintainable, offering efficient and scalable sensitivity analysis in complex groundwater modeling workflows.

## Introduction

The adjoint state method is a powerful analytical technique widely used in computational science to efficiently compute the sensitivities of model outputs to a large number of parameters. The method has also been widely applied in groundwater modeling across numerous areas, including sensitivity analysis (Sykes et al. 1985; Wilson and Metcalfe 1985; RamaRao et al. 1995; RamaRao and Mishra 1996; Skaggs and Barry 1996; Li and Yeh 1998; Cirpka and Kitanidis 2000; Jyrkama and Sykes 2006; Neupauer and Griebling 2012; Griebling and Neupauer 2013; Lu and Vesselinov 2015; RamaRao et al. 2017), parameter estimation (Neuman 1980; Sun and Yeh 1985, 1990; Townley and Wilson 1985; Lu et al. 1988; Yeh and Sun 1990; Lavenue and Pickens 1992; Yeh and Zhang 1996; Cardiff and Kitanidis 2008; Fienen et al. 2008; Wu et al. 2008), optimization (Ahlfeld et al. 1988; Tan et al. 2008), source identification (Neupauer and Wilson 1999, 2001; Michalak and Kitanidis 2004), and various other applications (see Sun 1994). Recently, the adjoint state method has been applied successfully to problems related to radionuclide transport in deep geological formations (Hayek et al. 2019, 2020, 2021).

A major advantage of the adjoint state method is its ability to compute sensitivities for all parameters at each model node in a single backward simulation, unlike the perturbation method, which requires separate forward simulations for each parameter. This approach greatly enhances computational efficiency, especially for large‐scale models with numerous parameters. The adjoint method is particularly well‐suited for generating sensitivity maps that highlight parameters with the most substantial impact on model outcomes. These maps are useful for building intuition regarding model behavior as well as for model calibration and uncertainty analysis, enabling modelers to concentrate on the most critical parameters. In addition, the adjoint method enables efficient computation of the full Jacobian matrix, where each of the m rows corresponds to one observation, and each row contains the partial derivatives with respect to the n parameters. Using adjoint‐based sensitivities, the Jacobian matrix can be assembled with m+1 model runs, one forward run and one adjoint run per observation, compared to m×n+1 runs required by the perturbation approach. This efficiency makes the adjoint method particularly attractive for integration with calibration tools such as PEST (Doherty 2015). Furthermore, since the gradient of a parameter‐estimation objective function can be obtained with only 1 forward model run and 1 adjoint solution, the adjoint state method is also widely used with the quasi‐Newton class of inversion algorithms, such as L‐BFGS (Liu and Nocedal 1989); see for example Oliver et al. (2008).

An earlier implementation of sensitivity computation was provided in MODFLOW‐2000 through the sensitivity equation method (Hill et al. 2000). This method calculates accurate derivatives by solving an additional sensitivity equation for each parameter. While this avoids the need for finite‐difference perturbations, the overall computational cost still scales with the number of parameters, similar to the perturbation method. In addition, MODFLOW‐2000 was difficult to maintain, lacked support for many modern packages, and could not keep pace with ongoing model development, which ultimately limited its long‐term adoption. These challenges underscore the need for more flexible and scalable approaches for sensitivity analysis in current‐generation groundwater models.

However, implementing the adjoint state method can be complex, often requiring extensive modifications to the forward model's source code to incorporate adjoint equations. This challenge is particularly pronounced for widely used software like MODFLOW 6 (MF6; Langevin et al. 2017), a groundwater flow (GWF) model where modifying the source code is intrusive and may affect compatibility with future software updates. Adjoint sensitivity capabilities were added to a previous version of MODFLOW (MODFLOW‐2005), as detailed in Clemo (2007). However, this implementation was intrusive and is incompatible with more recent MODFLOW versions, largely a result of a lack of maintenance of the adjoint version of MODFLOW‐2005 as new versions of MODFLOW‐2005 were released. This highlights a major challenge with implementing a general and widely applicable adjoint solver for codes with frequent releases.

In this work, we introduce “MF6‐ADJ”, a non‐intrusive, Python‐based implementation of the adjoint state method for MF6. Our approach uses the MODFLOW Application Programming Interface (MODFLOW API, Hughes et al. 2022), which provides the flexibility to control the time stepping process and extract solution components as the simulation advances. This approach to adjoint formulation and solution allows for a non‐intrusive implementation of the adjoint state method, avoiding any modifications to the MF6 source code. By doing so, we ensure that our code remains compatible with future versions of MF6, while retaining the computational efficiency and analytical precision of the adjoint method.

## Mathematical Background

The discrete forward model of MF6 is based on a generalized control‐volume finite‐difference (CVFD) approach in which a given cell can be connected to any number of surrounding cells. MF6 primary uses the conductance‐based formulation, where the flow between two cells is computed as the product of a hydraulic conductance and the head difference between the two cells. This conductance term ensures that the units of flow are consistent and intuitive. The substitution of the conductance‐based formulation into the mass balance equation gives the CVFD flow equation for cell n as follows (Langevin et al. 2017):

(1) $$\begin{array}{ll} & \sum_{m\in \eta_{n}}C_{n,m}h_{m}+\left(-\sum_{m\in \eta_{n}}C_{n,m}+P_{n}-\frac{SS_{n}A_{n}\Delta \upsilon_{n}}{t-t_{\mathrm{old}}}\right)h_{n}\\ & \;=-Q_{n}-\frac{SS_{n}A_{n}\Delta \upsilon_{n}}{t-t_{\mathrm{old}}}h_{n}^{t_{\mathrm{old}}}, \end{array}$$

where, $\eta_{n}$ is a list of cells connected to cell n, $C_{n,m}$ is the conductance between nodes n and m ($L^{2}T^{-1}$), $h_{m}$ and $h_{n}$ are the heads at nodes m and n at the current time‐step (L), $h_{n}^{t_{\mathrm{old}}}$ is the head at node n at the previous time‐step (L), t and $t_{\mathrm{old}}$ are the current and previous times (T), $P_{n}$ is a head coefficient used in the flow calculation ($L^{2}T^{-1}$), $Q_{n}$ is the head independent term used in the flow calculation representing any external sink or source term ($L^{3}T^{-1}$), $SS_{n}$ is the storage coefficient of cell n representing the volume of water that can be added or removed per unit volume of aquifer material per unit change in head in cell n ($L^{-1}$), $A_{n}$ is the horizontal cell area of cell n ($L^{2}$), and $\Delta \upsilon_{n}$ is the saturated thickness of cell n (L).

Equation (1) is formulated for each variable‐ and constant‐head cell in the grid. These equations can be assembled to be written in matrix form as follows:

(2) $$\left[A^{k}\right]h^{k}=b^{k},$$

where, $\left[A^{k}\right]=\left[A\left(\alpha^{k},h^{k}\right)\right]$ is the matrix of the coefficients that are a function of head at the current time‐step indexed by k, from the left side of Equation (1), for all active and constant head cells in the grid, called also the conductance matrix, and $b^{k}=b\left(\alpha^{k},h^{k-1}\right)$ is a vector of the constant terms for all active and constant head cells in the grid, composed of the terms of the right‐hand side (RHS) of Equation (1). Both, the matrix $\left[A^{k}\right]$ and the vector $b^{k}$ depend on the vector of input parameters which is denoted here by $\alpha^{k}$ (i.e., some of the input parameters might change over time, such as the injection/extraction rate, so we assign the time‐step index [*k*] to the parameter vector accordingly). The vector of the RHS depends also on the head at the previous time‐step indexed by (k−1). For many groundwater problems, Equation (1) is nonlinear in that individual entries in the conductance matrix $\left[A^{k}\right]$ are a function of head $h^{k}$ (the dependent variable). This is mainly the case for unconfined flow conditions, where the conductance depends on the water level in each cell. These nonlinearities are resolved through an iterative approach, involving repeated solutions of a linearized system of equations to handle nonlinear problems. At iteration k, the solution residual $r^{k}$ is written as:

(3) $$r^{k}=\left[A^{k}\right]h^{k}-b^{k}=0,$$

where $\left[A^{k}\right]$ and $b^{k}$ depend on the previous solution. For detailed information on the iterative procedure (i.e., Newton–Raphson formulation) and its implementation in MODFLOW 6, see Langevin et al. (2017).

### Model Sensitivities—Perturbation Method

The aim of sensitivity analysis is to examine how changes in each parameter (input) affect a performance measure (PM), typically through the first derivative of the PM with respect to the parameter. The general form of a PM, J, can be expressed as the sum of contributions $F^{k}$ from the individual time‐steps k:

(4) $$J=\sum_{k=1}^{N}F^{k}=\sum_{k=1}^{N}F\left(\alpha^{k},h^{k}\right),$$

where N is total number of time‐steps. Note that, in some cases, $F^{k}$ may be zero for some values of k. In practical applications, F could represent any function of the hydraulic head such as a combination of heads at specific locations and times, or an observation residual representing the differences between simulated and observed heads, or even the simulated hydraulic flux at a user‐defined interface at a combination of locations and times.

The sensitivity of J with respect to a given parameter $\alpha_{j}$ is measured by the first order derivative $\partial J/\partial \alpha_{j}$. These derivatives are often calculated using finite difference approximations, which involve perturbing model parameters and computing the resulting changes in model outputs through repeated forward simulations. This approach is herein referred to as the “pertubation method.” One drawback of these methods is that they may require a large number of forward flow simulations, which can be computationally demanding depending on model complexity, although such runs can be parallelized efficiently on computer clusters. The simplest approach is the forward finite difference scheme, which requires $N_{p}+1$ simulations to approximate all the sensitivities, where $N_{p}$ is the total number of input parameters. If the perturbation in the parameter is $\delta \alpha_{j}$ and $e_{j}$ is the $j^{\mathrm{th}}$ unit vector in ${\mathbb{R}}^{N_{p}}$ (having 1 in the $j^{\mathrm{th}}$ position and 0 elsewhere), then the forward finite difference scheme gives:

(5) $$\frac{\partial J}{\partial \alpha_{j}\;}=\frac{J\left(\alpha +\delta \alpha_{j}\;e_{j}\right)-J\left(\alpha \right)}{\delta \alpha_{j}\;}.$$

In addition to the computational cost of the forward finite difference scheme, truncation errors (from the finite step size) and rounding errors (from machine precision) can affect numerical accuracy. In practice, and consistent with established tools such as PEST (Doherty 2015) and UCODE (Poeter and Hill 1999), perturbing a parameter by about 1% of its value is often considered a sound choice.

### MODFLOW 6 Adjoint Sensitivity Model Equations

Unlike the perturbation method, which requires solving additional forward model run of type (2) and (3) for each input parameter, the adjoint state method computes sensitivities efficiently by solving a single set of adjoint equations alongside the forward model. This significantly reduces computational cost, especially for systems with numerous parameters and only a handful of PMs are of interest. For the MF6 model given by Equation (2) or Equation (3), the adjoint sensitivity coefficient of a PM given by Equation (4) with respect to parameter α is given by the following formula:

(6) $$\frac{\partial J}{\partial \alpha }=\sum_{k=1}^{N}\left[\frac{\partial F^{k}}{\partial \alpha }+{\lambda^{k-1}}^{T}\;\left(\frac{\partial \left[A^{k}\right]}{\partial \alpha }h^{k}-\frac{\partial b^{k}}{\partial \alpha }\right)\right],$$

where, $\left\{\lambda^{k}\right\}$ is the set of adjoint state vectors which are solutions of the following so‐called adjoint state system associated with the PM (4):

(7) $${\left[J^{k}\right]}^{T}\lambda^{k-1}={\left[\frac{\partial b^{k}}{\partial h^{k}}\right]}^{T}\lambda^{k}-\frac{\partial F^{k}}{\partial {h^{k}}^{T}},$$

with $\left[J^{k}\right]=\left[\frac{\partial r^{k}}{\partial h^{k}}\right]$, which is equal to the Jacobian matrix for nonlinear problems describing unconfined scenarios, and to the conductance matrix $\left[A^{k}\right]$ for linear problems characterizing confined flow conditions. The detailed mathematical derivations of Equations (6) and (7) are presented in the Appendix. Note that the symbol “J” is used for two distinct quantities: the italic J in Equation (4) denotes the scalar performance measure, while the bold matrix $\left[J^{k}\right]$ in Equation (7) denotes the Jacobian matrix. The difference in font style distinguishes these terms clearly.

The adjoint state system (7) is backward in time in the sense that it is solved successively for each time‐step starting from the last time‐step downwards for k=N−1,N−2,…,0 with the following “terminal” condition:

(8) $$\lambda^{N}=0.$$

System (7) shows that the adjoint state vectors are independent from the parameters and depend only on the performance measure.

An inspection of Equation (6) shows that the adjoint state vector is not just an auxiliary mathematical variable but holds physical significance. Indeed, if the parameter α is the injection/pumping rate at cell n and time‐step k, the direct application of Equation (6) to $\alpha =Q_{n}^{k}$ yields

(9) $$\frac{\partial J}{\partial Q_{n}^{k}}=\lambda_{n}^{k.}$$

Equation (9) states that the adjoint state variable at a given cell and time‐step represents the rate at which the performance measure varies per unit injection/pumping at that cell and time‐step. This interpretation originates from Wilson and Metcalfe (1985), who formulated it specifically for the steady‐state case.

One of the advantages of the adjoint state method is that the adjoint system is linear even for nonlinear forward problems. The matrix of the adjoint system is the transpose of that of the forward problem at the convergent iteration. The RHS depends on the PM, indicating that one adjoint system per PM type is required.

Note that, in Equation (4), F is a function of both the parameter vector $\alpha^{k}$ and the state variables $h^{k}$, which allows the inclusion of prior information or regularization terms that depend directly on parameter values. Since such regularization terms depend only on the parameters and not on the state variable (i.e., hydraulic head), they do not affect the adjoint state solution, which requires only derivatives of the performance measure with respect to head. Instead, the derivative of the regularization term with respect to the parameters appears in the expression for the sensitivity coefficients (first term on the right‐hand side of Equation 6, $\partial F^{k}/\partial \alpha$), representing the direct effect of prior information.

## MF6‐ADJ Code

### Implementation

**Figure 1:**
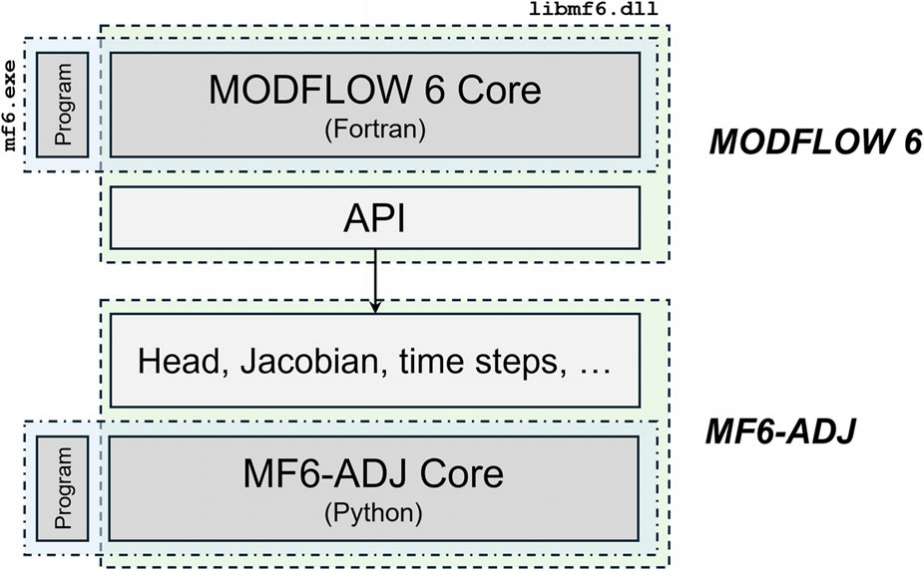
Overview of the implementation of adjoint methodology for MODFLOW 6.

The MF6‐ADJ code has been developed by implementing the generic adjoint methodology presented above for the MF6 code. The implementation has been carried out by using the MODFLOW API (Hughes et al. 2022). The MODFLOW API allows other programs to control MODFLOW and interactively change variables without having to modify the source code. With the MODFLOW API, there is no need to implement the adjoint methodology within the source code of MF6. The MODFLOW API facilitates run‐time access of all necessary information for solving the adjoint system (i.e., hydraulic head, saturation, Jacobian/conductance matrix, time stepping, etc.). Consequently, the adjoint methodology can be implemented externally using Python packages. In short, MF6‐ADJ uses the MODFLOW API to execute a single forward run of a MF6 GWF model, during which MF6‐ADJ stores the necessary solution components from MF6 and records these quantities in an HDF5 file (The HDF Group 2025). Once this forward run is completed, MF6‐ADJ no longer requires MF6 and solves for the user‐nominated PMs using only the HDF5 file. In this way, users can modify and/or add new performance measures without needing to re‐execute the forward model. Figure 1 presents an overview of the implementation of the adjoint sensitivity capability for MODFLOW 6.

**Figure 2:**
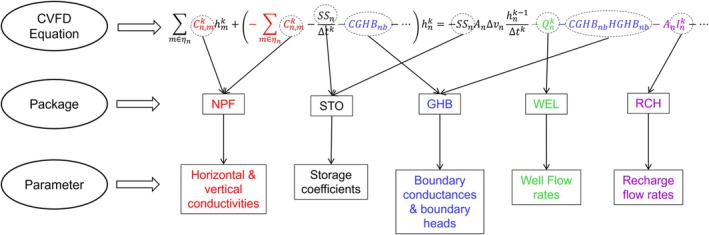
Overview of the parameter set used by each MODFLOW 6 package based on the CVFD equation. NPF is the Node‐Property Flow, which uses hydraulic conductivity through the hydraulic conductance. STO is the Storage package, which uses the storage coefficient and the specific yield. GHB is the General‐Head Boundary package, which uses the boundary conductance and the head assigned to the boundary condition. WEL is the Well package, which uses the injection/extraction flow rate. RCH is the Recharge package, which uses the recharge rate.

As shown in Equations (6) and (7), in addition to the derivatives of the conductance matrix with respect to the parameters, the computation of sensitivity coefficients by the adjoint state method involves the calculation of other derivatives. This includes derivatives of the RHS with respect to head and parameters and the derivatives of the PM with respect to head and parameters. These derivatives are analytically calculated using Python functions based on the mathematical expressions of the respective variables as defined in MF6. Each MF6 package uses certain types of parameters, which means some sensitivity parameters are specific to individual packages. Figure 2 illustrates, based on the CVFD equation, how each package uses specific types of parameters. For instance, the node property flow (NPF) package uses hydraulic conductivity which appears in the diagonal and off‐diagonal terms of the conductance matrix through the hydraulic conductance, and the storage (STO) package uses the storage coefficient which appears in the diagonal terms of $\left[A^{k}\right]$ and the RHS for the transient condition. Similarly, the General‐Head Boundary (GHB) package uses the boundary conductance which appears in the diagonal terms of $\left[A^{k}\right]$ and the RHS. For some packages such as the Well (WEL) and Recharge (RCH) packages, the parameters only appear in the RHS of the CVFD equation. For these parameters, the derivative of the conductance matrix is zero.

The current implementation supports several types of PMs such as one or more heads at one or more locations in space or time, sum‐of‐squared weighted head residuals (i.e. the $L^{2}$‐norm objective function used in most parameter estimation software), and certain boundary‐condition fluxes at one or more user‐defined locations in space and time. For example, the simulated surface‐water/groundwater exchange with one or more Streamflow‐Routing package (SFR) reaches at the end of each irrigation season. MF6‐ADJ currently supports several important MF6 parameters, including horizontal and vertical conductivities, storage coefficient, injection/extraction rate, recharge rate, boundary head, and boundary conductance.

The MF6‐ADJ code was designed around two primary classes: Mf6Adj and PerfMeas. Mf6Adj is a high‐level class that prepares the forward solution and processes the .adj input file (see subsection below). It performs the forward solve and stores the solution components required for the adjoint solve and writes the HDF5 file needed for PM‐specific adjoint calculations. This class is the only part of the code that interacts with MF6 and the MODFLOW API.

The PerfMeas class solves the adjoint system for each PM, calculates the requisite derivatives, and reports sensitivity analysis results. The PerfMeas class contains one or more PerfMeasRecord instances, representing discrete spatiotemporal information for each PM—specifically, for each model cell and stress period/time‐step where a user wants to analyze an aspect of the PM. The PerfMeas class reads solution components stored in a HDF5 file, processes it by solving the adjoint system backward in time for the current PM defined by the user‐defined PerfMeasRecord entries, and then writes the results to a new HDF5 file, which provides detailed and verbose outputs of the sensitivity analysis.

An example usage of MF6‐ADJ is as follows:

adj = mf6adj.Mf6Adj("test.adj", lib_name, verbose_level=1)
adj.solve_gwf()
adj.solve_adjoint()




We start by creating an instance of the Mf6Adj class, passing in the name of the performance measure file (in this case, “test.adj”), the relative path to the MODFLOW‐6 API library (lib_name), and, optionally, specifying the level of output detail. During initialization, the performance measure file is processed and checked. Next, MF6‐ADJ executes a single forward solve using MF6, which saves the solution components to an HDF5 file with the command solve_gwf(). At this point, solve_adjoint() loops through the unique PM listed in the performance measure file (“test.adj”), solving the adjoint system of equations for each PM backward in time while also recording results to a separate HDF5 file.

### Input File Organization

The MF6‐ADJ code uses a single performance measure file to specify the PMs of interest. In a similar fashion to the MF6 code, the performance measure file is organized into keyword blocks and is free format. A keyword block is a section of an ASCII input file that contains information about the PMs. A block begins with a line that starts with “begin performance_measure” followed by the name of the PM block and ends with the line “end performance_measure”. Within each block, the user specifies several records that define the PM, where each line corresponds to the information stored in a PerfMeasRecord instance. Each record line is formatted as follows:

SP TS cellid PMkey PMtype weight obsval


where SP, TS, cellid, PMkey, PMtype, weight, and obsval represent the stress period, time‐step, cell identification information, PM key (head or flux), PM type (direct or residual), weight, and observed value, respectively. For structured grid models, the cellid quantity should be the layer, row and column numbers, while for unstructured grid models, it should be layer, node number for DISV grids and the node number for DISU grids. The code supports two PM keys: head and flux, and two PM types: direct and residual. The PM key denotes whether a record pertains to a head or a flux and the PM type denotes whether a record is directly related to model outputs or is based on a weighted difference between the model output and an observed value. In this context, the flux PM key refers specifically to boundary fluxes (e.g., from GHB, RIV, SFR, or DRN packages) rather than intercellular flows, which is why only one cellid is needed in such records.

When multiple records are listed in a given PM block, the overall PM is defined as the sum of all heads/residuals/fluxes across the specified stress periods, time‐steps, and grid blocks.

The performance measure can be expressed as:

- Direct Performance Measure: The sum of all weighted heads or fluxes.
- Residual Performance Measure: The sum of squared weighted residual.

The observation residuals are defined as the squared weighted differences between calculated and observed values.

**Figure 3:**
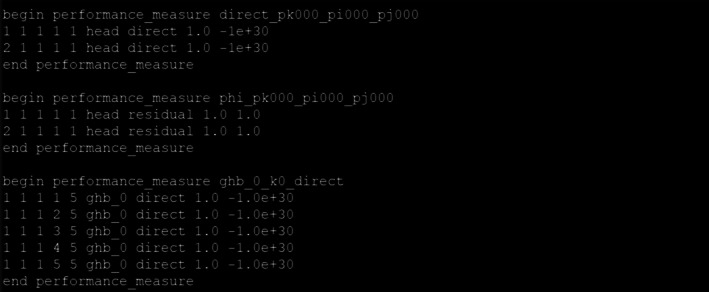
Example input file (performance measure file) of MF6‐ADJ.

Figure 3 shows an example of a MF6‐ADJ performance measure file. This file includes three PM blocks. The first block defines a PM as the sum of heads at grid block (1,1,1) during the first and second stress periods. The second block represents the head residual at grid block (1,1,1) over the same periods, while the third block specifies the GHB flux across the fifth column interface during the first stress period; this GHB‐based PM indicates we are interested in the sensitivity of the model inputs to this boundary flux.

## Example Applications

MF6‐ADJ has been tested and validated against analytical solutions and numerical sensitivities obtained using the perturbation method. The main purpose is to test the implementation of MF6‐ADJ using several scenarios including structured/unstructured grids and confined/unconfined flow conditions using various types of performance measures and MF6 GWF models. Unless otherwise noted, all simulations and analyses were performed on a Dell Precision 7670 laptop running Windows 11, equipped with an Intel Core i9 processor, and 64 GB of RAM. The execution times reported for the MF6 forward run, perturbation method, and MF6‐ADJ were obtained on this system.

### Comparison with Analytical Solution

Numerical adjoint states calculated from MF6‐ADJ are compared against analytical results obtained from Lu and Vesselinov (2015). We consider a specific case where the domain is a rectangle measuring 500m×400m, with fixed heads at the lateral boundaries, h=1 m at the left (x=0) and h=0 m at the right (x=500 m), and no‐flow boundaries at the bottom and top (y=0 and y=400 m). A constant hydraulic conductivity of 10 m/d is used. The MODFLOW 6 numerical grid consists of a single layer, 100 rows and 80 columns, each cell being 5 m by 5 m in size, and the Constant‐Head (CHD) package is used to define the boundary conditions. A single stress period of 1 day with one time‐step is used to simulate steady state conditions.

**Figure 4:**
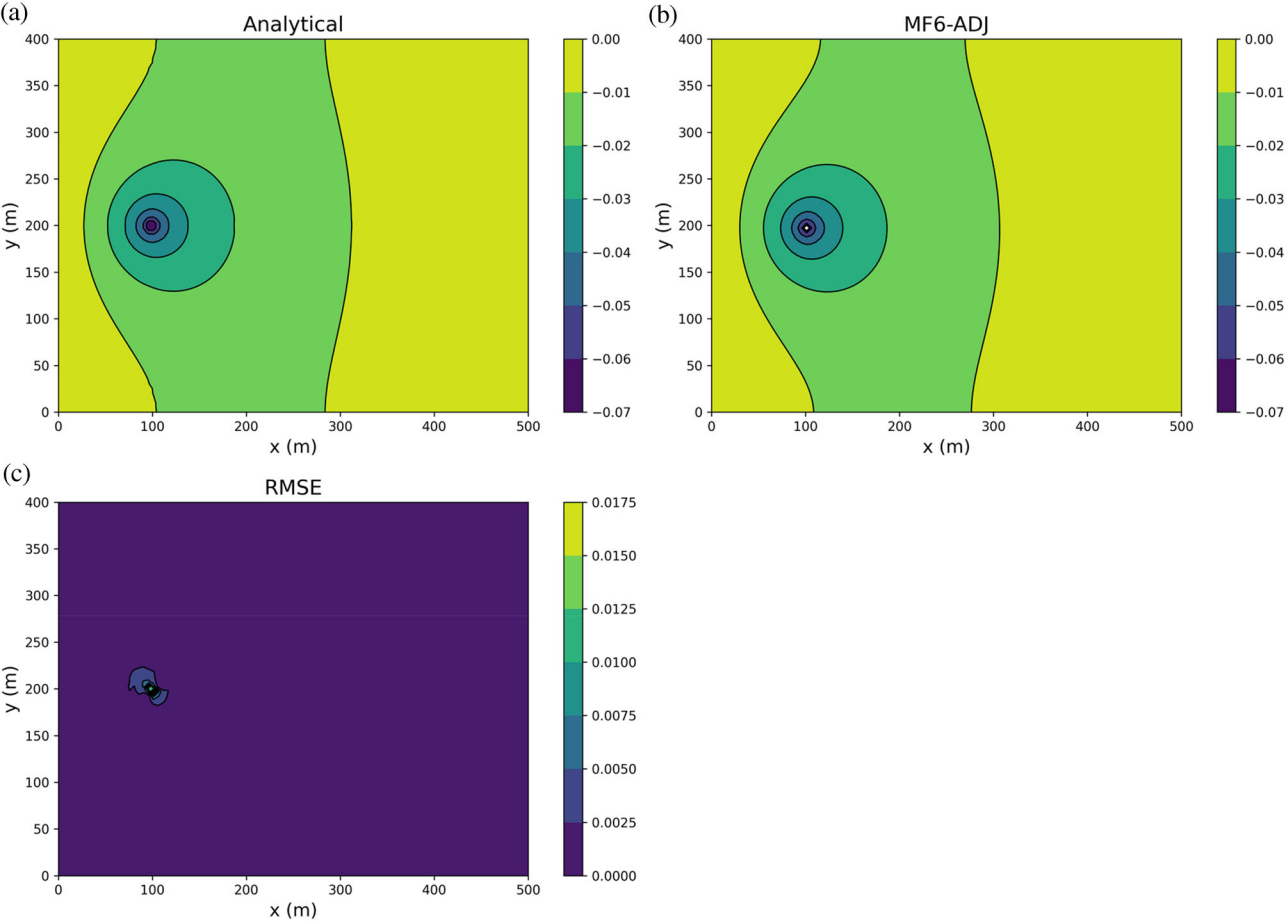
Contour maps of the analytical (a) and MF6‐ADJ (b) adjoint state variable λ [d/m^2^] associated with a head PM defined at location (100 m, 200 m). Higher absolute values of λ are observed in the vicinity of this location, with nearly circular contours indicating that pumping or injection near the observation cell exerts the strongest influence on the measured head. Panel (c) shows the absolute difference between the analytical and MF6‐ADJ solutions at each location, highlighting that the largest discrepancies occur primarily near the well.

For the adjoint state simulation, we use a performance measure defined as the head at the point (100 m, 200 m). Adjoint state contour maps, obtained both analytically and numerically using MF6‐ADJ, are shown in Figure 4a and 4b. The analytical results are derived from equation (42) of Lu and Vesselinov (2015). As indicated in Equation (9), the adjoint state at a given cell and time‐step represents the sensitivity of the performance measure to the injection/pumping rate at that cell and time‐step. Here, the performance measure is the head [m] and the unit of the pumping rate is [m^3^/d], resulting in adjoint state units of [d/m^2^]. The highest adjoint state values (in absolute value) occur in the vicinity of the cell where the performance measure is defined, with nearly circular contour lines centered on that location, as expected because pumping or injection near that cell has the greatest influence on the head measured there.

Figure 4c shows the spatial distribution of the absolute difference between the analytical and MF6‐ADJ results at each location, which effectively represents the root mean square error (RMSE) for this steady‐state case. The RMSE map highlights that the largest differences are located primarily near the well, while the agreement remains excellent farther from the well. The overall RMSE was calculated to be 6 × 10^−4^ d/m^2^, confirming excellent agreement between analytical and numerical solutions. It should be noted that this error reflects the combined accuracy of both the MF6 numerical solution and the Python‐based linear solver used for the adjoint state solution. A looser solver tolerance in MF6 may result in less accurate heads and therefore larger errors, while a looser tolerance in the Python solver may also elevate errors despite an accurate MF6 solution. Thus, maintaining sufficiently strict solver tolerances in both components is important to ensure reliable sensitivity estimates.

### Synthetic Model with Nested Grid

The objective of this test is to verify the implementation when the coefficients of the conductance matrix explicitly depend on the parameters used to calculate sensitivities, such as hydraulic conductivity. Cell hydraulic conductivities appear in the conductance matrix through the conductance terms, and calculating their sensitivities requires computing the derivative of the conductance matrix with respect to the cell's conductivity (see Equation 6). For each cell, this involves iterating over all its connections. To ensure robustness of our implementation, an unstructured grid (DISV grid) is used for this verification.

**Figure 5:**
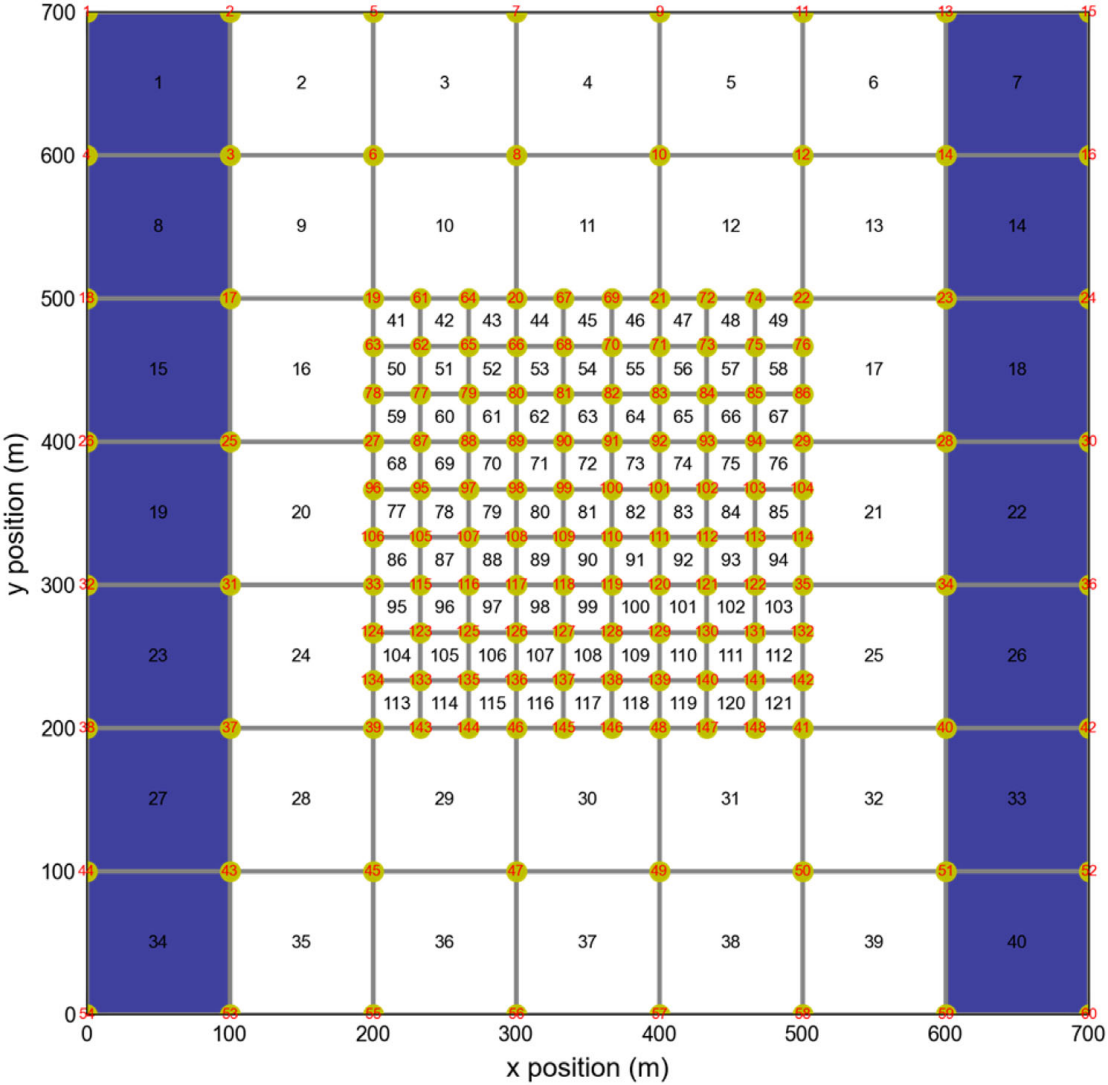
Model grid used for the nested grid problem. General‐head boundary cells are marked in blue. Cell numbers are shown inside each model cell. Vertices are also numbered and are shown in red. The PM is the head at cell 80.

The problem setup consists of an unstructured, nested grid as illustrated in Figure 5. The outer grid has cells measuring 100 m on each side, while the nested grid contains cells with sides that are one‐third this size. Cells and vertices are numbered as shown in Figure 5. The model assumes a uniform top elevation of 0 m and a uniform bottom elevation of −100 m. Varying boundary conditions are applied on the left and right sides using the GHB package such that GWF is from right to left. The model consists of 121 cells. A single steady‐state stress period of 1 d is simulated, starting with an initial head of zero. The hydraulic conductivity is assumed to be heterogeneous and follows a log‐normal distribution.

**Figure 6:**
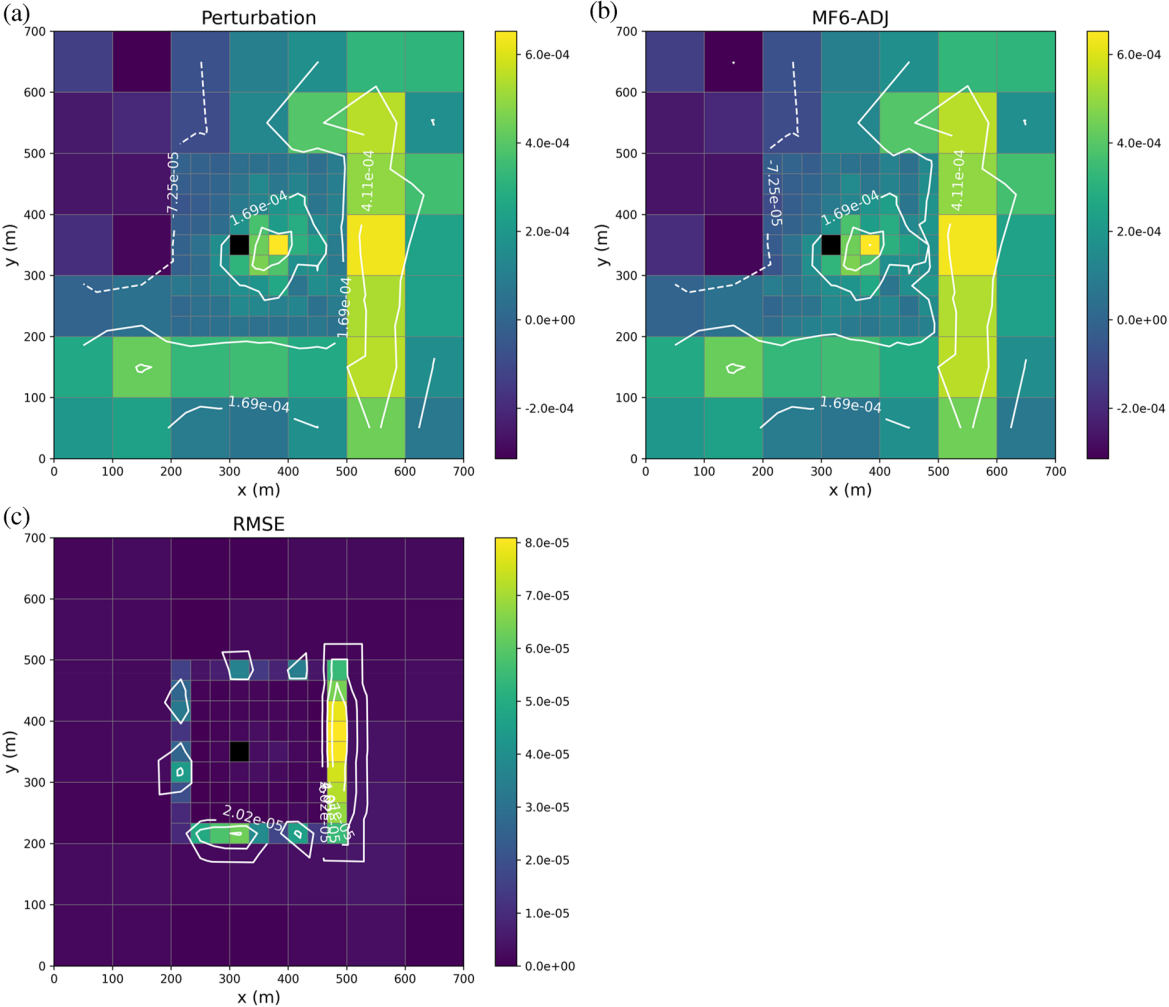
Sensitivity maps of the hydraulic head with respect to horizontal hydraulic conductivity, ∂h/∂K, for the nested grid problem. Panels (a) and (b) show the perturbation and MF6‐ADJ calculated sensitivities, respectively. Contour lines and color shading represent the magnitude of the sensitivity with units of [m/(m/d)], that is, head change per unit change in hydraulic conductivity. Panel (c) shows the absolute difference (RMSE) between the perturbation and MF6‐ADJ results, highlighting that the largest discrepancies occur in the small cells located at the interface between the refined central grid and the surrounding coarser grid.

For this test case, the piezometric head at cell 80 is chosen as the performance measure. Sensitivity coefficients are computed using the perturbation method and the developed MF6‐ADJ code. Figure 6 shows the sensitivity coefficient maps of the performance measure with respect to horizontal conductivity; that is, the maps represent sensitivities of head to changes in horizontal conductivity. The spatial patterns observed in the sensitivity maps are influenced by the heterogeneity of horizontal conductivity, which follows a log‐normal distribution in this example. However, the general pattern is that horizontal conductivity sensitivities upgradient of the performance measure have a positive sign, indicating that increasing horizontal conductivity in these areas leads to an increase in the simulated performance measure groundwater level, while increasing horizontal conductivity downgradient of the performance measure leads to a decrease in simulated groundwater level performance measure (a negative relation). Panels (a) and (b) show the perturbation‐based and MF6‐ADJ sensitivity maps, respectively, with overall good agreement between the two approaches. Panel (c) presents the absolute difference (RMSE) between the two maps, which reveals that the largest discrepancies occur in the small cells located at the interface between the refined central grid and the surrounding coarser grid. This is likely due to resolution effects at the nested grid boundary. The calculated overall RMSE between MF6‐ADJ and perturbation sensitivities is $2.35\times 10^{-5}d$ (i.e., m/[m/d]), indicating excellent consistency. The perturbation method (panel a) required 122 forward runs (one base case run and 121 perturbation runs, each corresponding to a perturbed parameter). In contrast, the MF6‐ADJ approach (panel b) required only two runs: one forward run and one adjoint run.

### Freyberg Model

**Figure 7:**
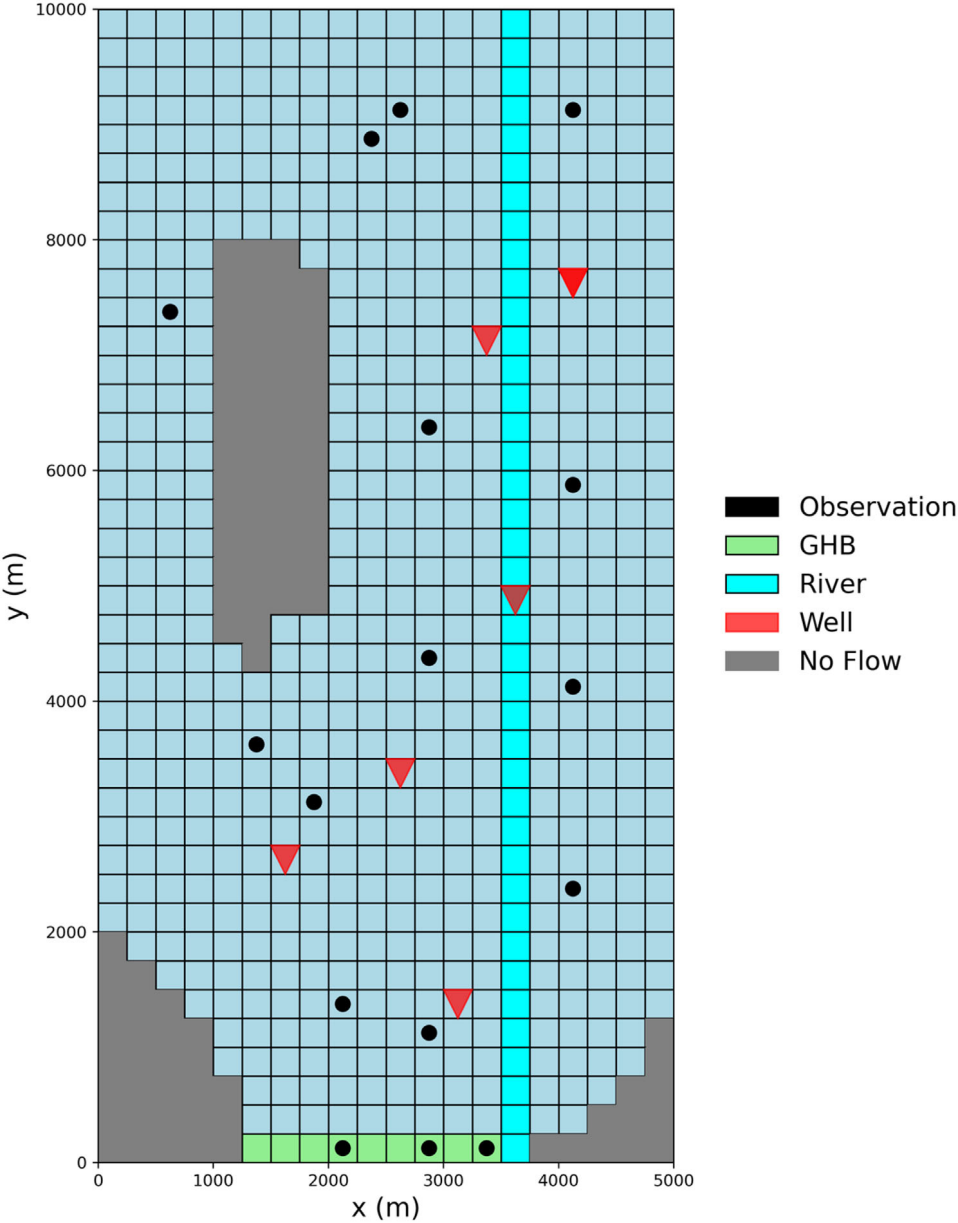
Schematic representation of Freyberg model. The grid consists of 705 active cells, with inactive regions shown in gray. General‐Head Boundary (GHB) cells are depicted in light green at the bottom, while Streamflow‐Routing (SFR) cells are displayed in cyan. Observation points are marked by black circles, and the locations of pumping wells are indicated by red triangles.

In this test case, we use the hypothetical aquifer system, the Freyberg model (Freyberg 1988), as revised by Hunt et al. (2019). The numerical model consists of a single‐layer, shallow, water‐table aquifer with no‐flow boundaries on the bottom and north‐east–west, while the southern boundary features a GHB (Figure 7). The MF6 grid has 40 rows and 20 columns, with each cell measuring 250 m on a side. Inactive outcrop zones are present within the grid, along with a straight river running along column 15. The model includes six pumping wells and assumes a spatially uniform recharge rate (R). Heads were observed at sixteen locations (Figure 7). The model simulates a two‐year period with 25 stress periods: an initial single steady‐stress period lasting 1 day, followed by 24 transient monthly stress periods, with one time‐step per period. Bottom elevations are not uniform but are relatively flat on the east side and gently slope toward the south and west. No‐flow cells, representing impermeable outcrop zones, are present in the western and southeastern corners of the model, resulting in a total of 705 active cells in the model. A constant horizontal hydraulic conductivity of 1 m/d and a specific storage coefficient of 10^−6^ 1/m were assigned to all active cells.

For this test case, the performance measure of interest is the sum‐of‐squared weighted differences (residuals) between simulated and observed heads at all observation points across all time‐steps; this PM demonstrates how MF6‐ADJ can be used in conjunction with parameter estimation software to map the sensitivity of the objective function with respect to important model inputs. The forward MF6 run takes approximately 10 s to complete. Sensitivity maps of recharge rates were calculated using both MF6‐ADJ and the perturbation method. The adjoint solve takes around 40 s. The recharge rate was applied to all active cells of the model and is time‐dependent, resulting in a total of 17,625 parameters (705 cells × 25 time‐steps).

**Figure 8:**
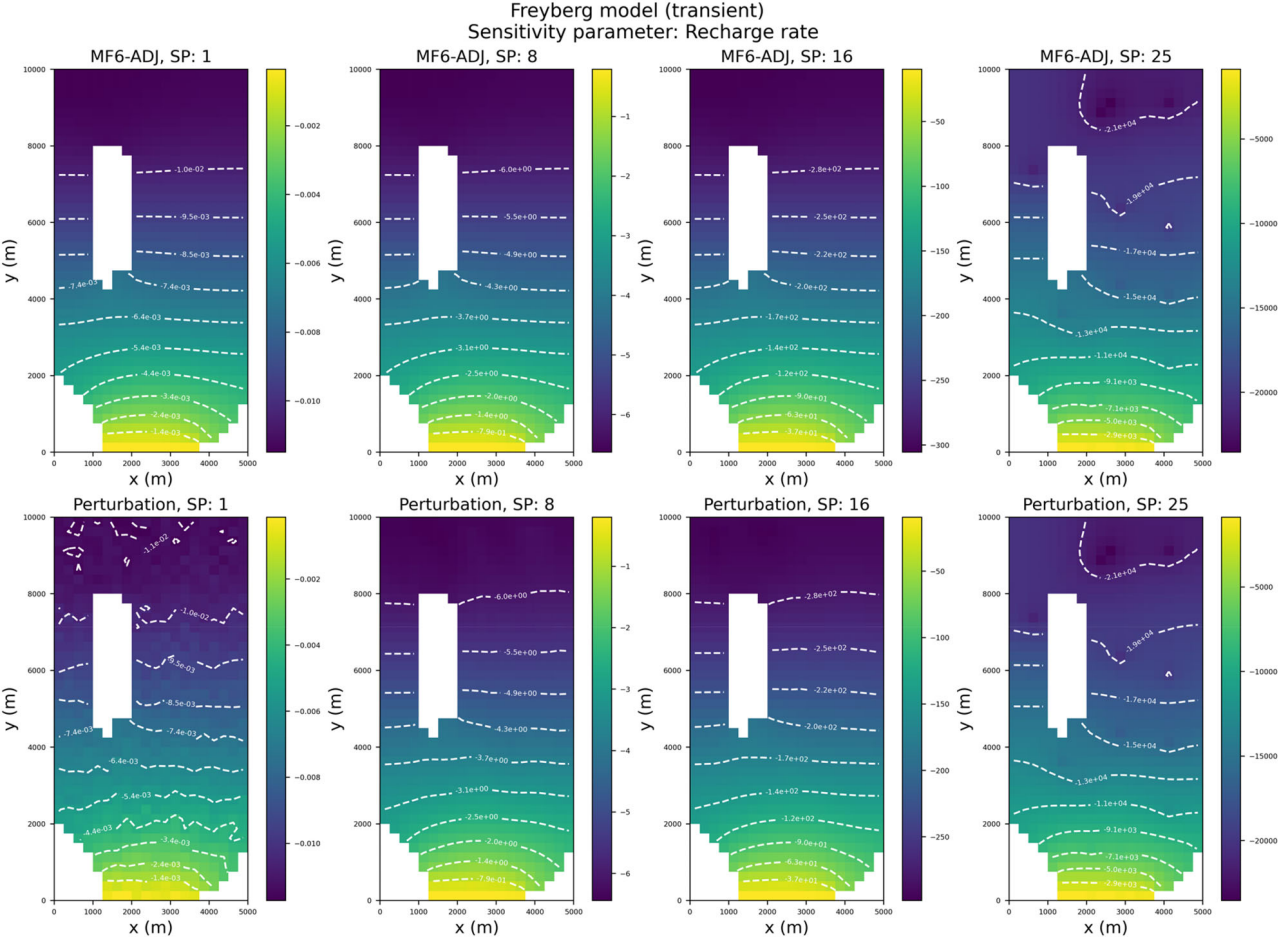
Comparison between contour sensitivity maps of recharge rate obtained from MF6‐ADJ (top) and calculated by the perturbation method (bottom) at four stress periods: 1, 8, 16, and 25.

Figure 8 compares the recharge rate sensitivity maps obtained by MF6‐ADJ (top) and the perturbation method (bottom) for selected stress periods. Perturbation results were generated using a 1% increment for each parameter. Overall, the results from both methods are in good agreement. Generating sensitivity maps for all stress periods using MF6‐ADJ took less than 1 min, whereas the perturbation method required resolving 17,626 forward runs, which took approximately 2 d of simulation time. These timings were obtained from serial runs. This demonstrates the computational efficiency of the adjoint‐state method.

The perturbation results displayed irregularities (such as oscillating contour lines, particularly for the first stress period). While solver convergence difficulties could also introduce artifacts, such issues would be expected to manifest similarly in the adjoint‐state results, which is not the case. Instead, our observations suggest that the irregularities are more likely attributable to the finite‐difference approximation inherent in the perturbation method and the choice of perturbation increment. If the increment is too small, numerical noise is amplified; if too large, nonlinear effects dominate, both leading to inaccuracies. In contrast, the adjoint‐state method provides analytical derivatives that remain smooth and stable, yielding more accurate results. This makes it more suitable for calculating sensitivity coefficients, not only due to its computational efficiency but also because of its precision. Accurate derivative calculations are also essential in contexts where sensitivities feed into parameter estimation, data assimilation, or uncertainty analysis as numerical noise in derivatives can adversely affect convergence and stability in such procedures (Doherty 2015).

### San Pedro Regional Model

For many groundwater studies, estimating stream depletion is an important objective, particularly when an aquifer is hydraulically connected to a nearby stream, as pumping from a well can draw water both from aquifer storage and the stream. Various analytical and numerical methods exist for estimating this depletion if the well's location is known. However, if multiple potential well locations are considered, calculating stream depletion for each can be computationally intensive, especially if the number of potential well locations is large or the model execution time is large (Neupauer and Griebling 2012; Griebling and Neupauer 2013; Ou et al. 2016). The adjoint approach is an efficient alternative which enables stream depletion estimates across any well location with just one additional simulation. In this section, we use MF6‐ADJ to calculate stream depletion associated with the Upper San Pedro Basin.

The Upper San Pedro Basin model was developed by Leake et al. (2014) to simulate surface water/groundwater interactions. The model includes the San Pedro River, which supplies natural recharge to the aquifer and is essential for local ecosystems and communities. The model has 440 rows and 320 columns, with a constant grid size of 250 m. The model has 5 layers through which GWF is primarily horizontal through laterally extensive hydrogeologic units. Layer 1 represents alluvium along the San Pedro River and ranges from 3 to 100 m thick. Layers 2 and 3 represent silt and clay, varying from 10 to 180 m. Layer 4 is very permeable, consists of sand, gravel, and conglomerate, and is up to 400 m thick. Layer 5 covers the entire area, represents limestone and sedimentary rock aquifers, and has a maximum thickness of 1500 m. The model has a total of 115,531 active cells. The GHB package is used to represent lateral exchanges along the north‐east of the model in layers 4 and 5. For more information on the model setup, boundary conditions, and parameters, see Leake et al. (2014).

The SFR package is used to simulate surface‐water/groundwater interactions. In this application, the performance measure is stream depletion, defined as the sum of surface water/groundwater fluxes between groundwater and the SFR network across all reaches and simulation times (a more discrete PM related to this flux can easily be constructed). In addition to the pumping rate at a specific well, the system parameters also include horizontal and vertical hydraulic conductivity. We aim to calculate sensitivity maps for the change in surface‐water/groundwater exchange to changes in pumping rates for all active model cells, as well as for the horizontal and vertical hydraulic conductivity. The sensitivity of stream depletion with respect to the pumping rate at a specific well indicates the proportion of water drawn from the river compared to the subsurface groundwater. We note that some subsurface water may also originate from GHB cells included in the model, so the stream depletion sensitivity reflects contributions from the river, groundwater storage, and GHB inflows. Using the adjoint state method, this sensitivity is obtained directly as the adjoint state at the specific location (see Equation 9). That is, the adjoint state in a cell, associated with the specified surface‐water/groundwater PM, is equivalent to the capture fraction defined by Leake et al. (2014).

**Figure 9:**
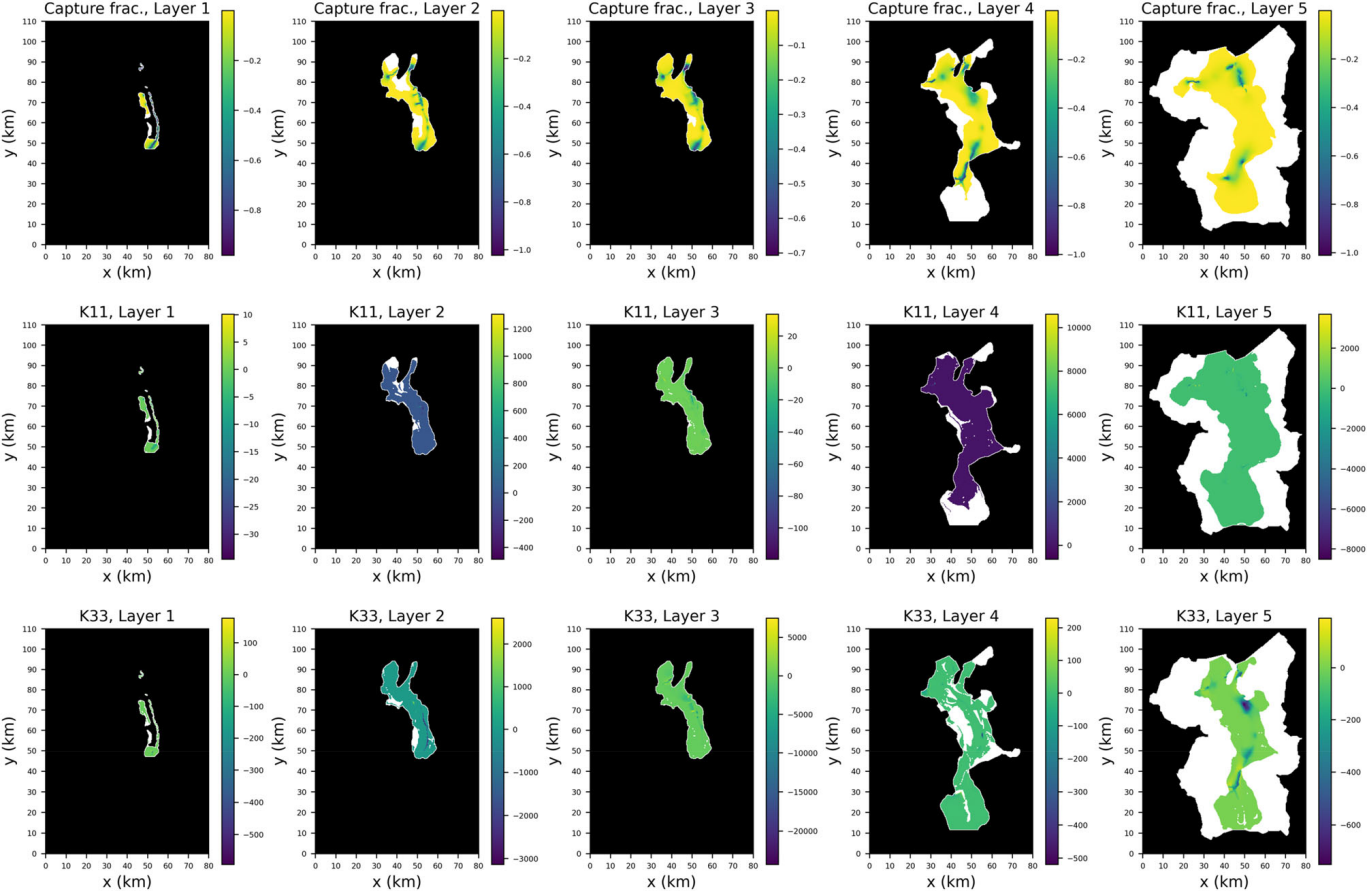
Sensitivity maps for San Pedro model calculated for all layers using MF6‐ADJ. Top: capture fraction, middle: horizontal hydraulic conductivity (K11), bottom: vertical hydraulic conductivity (K33). Inactive regions shown in black.

For this test case, the forward simulation is transient with a 10‐year simulation period, discretized into a single stress period with 10 one‐year time‐steps. The forward simulation takes approximately 10 s, while the adjoint run takes around 22 s. Figure 9 shows the sensitivity maps associated with the Upper San Pedro model calculated for all layers using MF6‐ADJ. In this figure, the first row displays the capture fractions (Leake et al. 2010), representing stream depletion (i.e., the ratio of capture rate to pumping rate). The second row shows sensitivity maps for horizontal hydraulic conductivity, and the third row shows those for vertical hydraulic conductivity. Sensitivity values below 10^−4^ are shown in white. As expected, capture is high for pumping locations near river reaches across all layers, while locations further from these reaches exhibit lower capture. Capture fractions are negative and indicate that an injection of water decreases river depletion. Sensitivity to horizontal hydraulic conductivity shows a relatively uniform distribution across the area, whereas vertical hydraulic conductivity sensitivity patterns resemble those of the capture fraction maps.

The sensitivity maps in Figure 9 were generated by performing one forward run and one adjoint run, requiring about 30 s of wall time. In contrast, using a perturbation‐based approach would necessitate solving the forward model 3×(115,531+1)=346,596 times. When run in serial, this corresponds to approximatively 40 days of wall time, demonstrating the efficiency of our MF6‐ADJ code compared to the perturbation method.

## Limitations

The initial release of MF6‐ADJ does not yet support all advanced MF6 GWF functionality. Currently, unsupported packages include:

- Multi‐aquifer Well (MAW) Package.
- Water Mover (MVR) Package.
- Skeletal Storage, Compaction, and Subsidence (CSUB) Package.
- Unsaturated Flow (UZF) Package.

In addition, MF6‐ADJ is currently limited to the GWF model. It does not yet support other model types in MODFLOW 6, such as the Groundwater Transport (GWT) model. Extending the adjoint framework to these capabilities would require substantial further development.

Future development of MF6‐ADJ will be guided by user feedback. Users are encouraged to submit enhancement requests, including supporting new parameters, additional MF6 packages, or new PM keys and types, through the MF6‐ADJ GitHub repository.

In addition to package‐level limitations, users should be aware that the use of Constant‐Head (CHD) boundaries in MF6 models has important implications for sensitivity and adjoint‐state analyses. CHD cells enforce a fixed head value, which leads to a zero adjoint state at those locations. This behavior is expected, as a fixed head implies no variation in response with respect to model parameters at that node. In contrast, GHB conditions allow head values to vary based on model parameters, leading to nonzero adjoint states and enabling meaningful sensitivity information. While CHDs are conceptually valid and commonly used, they can mask sensitivities in the regions where they are applied. For this reason, users are encouraged to convert CHD boundaries to GHB boundaries where feasible, especially when sensitivity analysis or parameter estimation is a primary goal. In MF6, this conversion is relatively straightforward and involves specifying a conductance value and tagging the boundary as GHB. Making this adjustment can prevent the misinterpretation of sensitivity results or the underestimation of parameters influence near model boundaries.

Another important consideration is the computational scaling of the adjoint‐state method with respect to the number of PMs. Each PM requires the solution of a separate adjoint system, which means that the overall cost increases linearly with the number of PMs. Consequently, MF6‐ADJ provides the greatest advantage in applications where the number of parameters is large but the number of PMs is relatively small, such as when a modeler is interested in a limited set of observation heads or fluxes. In contrast, when hundreds or thousands of PMs are considered simultaneously, the computational benefits of the adjoint approach compared to perturbation‐based sensitivity methods diminish, and the choice of approach should be guided by the relative balance between parameter and observation counts. Users are encouraged to weigh these trade‐offs when designing sensitivity or data‐assimilation studies, and to consider whether the adjoint or perturbation‐based approaches provide the more efficient computational path for their specific application.

## Conclusion

In this paper, we presented MF6‐ADJ, an adjoint sensitivity analysis tool developed for MF6. MF6‐ADJ provides a robust, efficient, and non‐intrusive solution for conducting sensitivity analysis in groundwater models. Unlike traditional methods that require direct modifications to the MF6 source code, MF6‐ADJ uses the MODFLOW API to externally implement the adjoint sensitivity analysis. This approach ensures compatibility with ongoing and future developments in MF6 without necessitating maintenance of adjoint sensitivity changes to its code base.

Using the adjoint state method, MF6‐ADJ computes sensitivity coefficients by solving a single set of adjoint equations in addition to the forward model. This approach reduces computational costs, particularly for models with numerous parameters, compared to the perturbation method, which requires extra forward model simulations for each parameter. Consequently, the adjoint‐based MF6‐ADJ method enhances the speed and efficiency of sensitivity analysis, offering clear advantages for models with extensive parameter spaces, while the benefit decreases when the number of performance measures is large.

The MF6‐ADJ implementation is particularly beneficial for real‐world applications, enabling detailed mapping of sensitivity coefficients across the computational grid. This facilitates the efficient assessment of local sensitivities throughout the model domain, aiding in parameter optimization, stream depletion estimation, and groundwater management planning. By supporting key parameters across various MODFLOW packages, such as hydraulic conductivities, storage coefficients, recharge rates, and boundary conditions, MF6‐ADJ is a comprehensive tool adaptable to diverse hydrogeological conditions.

The successful validation of MF6‐ADJ against analytical solutions and alternative numerical methods confirms its reliability and accuracy. These validations also underscore its ability to provide computational efficiency and precision in parameter sensitivity analysis, positioning MF6‐ADJ as a valuable asset in groundwater modeling and resource management.

## Authors' Note

The authors do not have any conflicts of interest or financial disclosures to report.

## Data Availability

The code MF6‐ADJ is freely available at https://github.com/INTERA‐Inc/mf6adj. Several example Jupyter notebooks are also provided, including demonstrations of using MF6‐ADJ with the Freyberg and San Pedro models. Community contributions are welcome.

## Derivation of Adjoint sensitivity equations of MODFLOW 6

We write the system of equations solved by MF6 at each time‐step k as a solution residual of the form:

(A.1)
$$r^{k}=\left[A^{k}\right]h^{k}-b^{k}=0.$$

Note that the solution residual depends on the vector of hydraulic head at the current and last time‐steps as well as the parameter vector: $r^{k}=r^{k}\left(\alpha^{k},h^{k},h^{k-1}\right)$.

We introduce a set of (yet arbitrary) vectors $\left\{\lambda^{0},\lambda^{1},\ldots ,\lambda^{N}\right\}$ each has the same dimension as $h^{k}$. These vectors are called the adjoint state vectors which will be determined later. Multiplying (A.1) by ${\lambda^{k-1}}^{T}$ and summing over all time‐steps, we get after adding the obtained summation to (4):

(A.2)
$$J=\sum_{k=1}^{N}F^{k}+\sum_{k=1}^{N}{\lambda^{k-1}}^{T}\;r^{k}.$$

The derivative of J with respect to a given sensitivity parameter α can be written as follows:

(A.3)
$$\begin{array}{ll} \frac{\partial J}{\partial \alpha } & =\sum_{k=1}^{N}\left(\frac{\partial F^{k}}{\partial \alpha }+\frac{\partial F^{k}}{\partial h^{k}}\frac{\partial h^{k}}{\partial \alpha }\right)+\sum_{k=1}^{N}{\lambda^{k-1}}^{T}\\ & \;\times \left(\frac{\partial r^{k}}{\partial \alpha }+\left[\frac{\partial r^{k}}{\partial h^{k}}\right]\frac{\partial h^{k}}{\partial \alpha }+\left[\frac{\partial r^{k}}{\partial h^{k-1}}\right]\frac{\partial h^{k-1}}{\partial \alpha }\right). \end{array}$$

Using (A.1) to calculate $\partial r^{k}/\partial \alpha$, Equation (A.3) can be rewritten as:

(A.4)
$$\begin{array}{ll} \frac{\partial J}{\partial \alpha } & =\sum_{k=1}^{N}\left[\frac{\partial F^{k}}{\partial \alpha }+{\lambda^{k-1}}^{T}\;\left(\frac{\partial \left[A^{k}\right]}{\partial \alpha }h^{k}-\frac{\partial b^{k}}{\partial \alpha }\right)\right]\\ & +\sum_{k=1}^{N}\left(\frac{\partial F^{k}}{\partial h^{k}}+{\lambda^{k-1}}^{T}\left[\frac{\partial r^{k}}{\partial h^{k}}\right]\right)\frac{\partial h^{k}}{\partial \alpha }\\ & +\sum_{k=1}^{N}{\lambda^{k-1}}^{T}\left[\frac{\partial r^{k}}{\partial h^{k-1}}\right]\frac{\partial h^{k-1}}{\partial \alpha }. \end{array}$$

The last term of the RHS of Equation (A.4) can be rewritten as follows:

(A.5)
$$\sum_{k=1}^{N}{\lambda^{k-1}}^{T}\left[\frac{\partial r^{k}}{\partial h^{k-1}}\right]\frac{\partial h^{k-1}}{\partial \alpha }=-\sum_{k=1}^{N}{\lambda^{k}}^{T}\left[\frac{\partial b^{k}}{\partial h^{k}}\right]\frac{\partial h^{k}}{\partial \alpha }.$$

In Equation (A.5), we have assumed that $\lambda^{N}=0$ and that $h^{0}$ (the initial head) is independent from α. Moreover, we have used (A.1) to replace $\partial r^{k+1}/\partial h^{k}$ by $-\partial b^{k}/\partial h^{k}$. We note that the condition $\lambda^{N}=0$ is necessary for the resolution of the adjoint system shown below. Under this condition, Equation (A.4) becomes:

(A.6)
$$\begin{array}{ll} \frac{\partial J}{\partial \alpha } & =\sum_{k=1}^{N}\left[\frac{\partial F^{k}}{\partial \alpha }+{\lambda^{k-1}}^{T}\;\left(\frac{\partial \left[A^{k}\right]}{\partial \alpha }h^{k}-\frac{\partial b^{k}}{\partial \alpha }\right)\right]\\ & +\sum_{k=1}^{N}\left(\frac{\partial F^{k}}{\partial h^{k}}+{\lambda^{k-1}}^{T}\left[\frac{\partial r^{k}}{\partial h^{k}}\right]-{\lambda^{k}}^{T}\left[\frac{\partial b^{k}}{\partial h^{k}}\right]\right)\frac{\partial h^{k}}{\partial \alpha }. \end{array}$$

Since $\left\{\lambda^{k}\right\}$ are arbitrary, they can be chosen to eliminate state sensitivity vectors $\partial h^{k}/\partial \alpha$ from Equation (A.6). This can be obtained by setting the term multiplied by $\partial h^{k}/\partial \alpha$ in (A.6) to zero. Therefore, the sensitivity coefficient simplifies to:

(A.7)
$$\frac{\partial J}{\partial \alpha }=\sum_{k=1}^{N}\left[\frac{\partial F^{k}}{\partial \alpha }+{\lambda^{k-1}}^{T}\;\left(\frac{\partial \left[A^{k}\right]}{\partial \alpha }h^{k}-\frac{\partial b^{k}}{\partial \alpha }\right)\right],$$

with the vectors $\left\{\lambda^{k}\right\}$ are solutions of the so‐called adjoint state system given by:

(A.8)
$${\left[J^{k}\right]}^{T}\lambda^{k-1}={\left[\frac{\partial b^{k}}{\partial h^{k}}\right]}^{T}\lambda^{k}-\frac{\partial F^{k}}{\partial {h^{k}}^{T}}.$$

where $\left[J^{k}\right]=\left[\frac{\partial r^{k}}{\partial h^{k}}\right]$. For nonlinear problems (i.e. unconfined conditions), the matrix $\left[J^{k}\right]$ is the Jacobian matrix, while it is simply the conductance matrix $\left[A^{k}\right]$ for linear scenarios (i.e. confined conditions).

Note that, the adjoint system (A.8) is backward in time which is solved with the terminate condition $\lambda^{N}=0$.
